# Individualized Diets in Patients with Kidney Disease and Kidney Transplants: A Narrative Review

**DOI:** 10.3390/life15060896

**Published:** 2025-05-31

**Authors:** Lilio Hu, Greta Borelli, Elisa Gessaroli, Chiara Ruotolo, Sofia Bin, Giuliana Papalia, Gemma Patella, Maria Elena Liberti, Olga Baraldi, Gianluigi Zaza, Irene Capelli, Michele Provenzano

**Affiliations:** 1 Department of Medical and Surgical Sciences (DIMEC), Alma Mater Studiorum, University of Bologna, 40138 Bologna, Italy; greta.borelli@studio.unibo.it (G.B.); elisa.gessaroli4@studio.unibo.it (E.G.); sofiabin8@gmail.com (S.B.); olga.baraldi3@unibo.it (O.B.); irene.capelli4@unibo.it (I.C.); 2 Nephrology and Dialysis Unit, Santa Maria Delle Croci Hospital, AUSL Romagna, 48121 Ravenna, Italy; 3 Division of Nephrology, University of Campania “Luigi Vanvitelli”, 80138 Naples, Italy; chiara.ruotolo@yahoo.it (C.R.); m.elenaliberti@libero.it (M.E.L.); 4 Nephrology, Dialysis and Renal Transplant Unit, Department of Pharmacy, Health and Nutritional Sciences, University of Calabria, Rende-Hospital ‘SS. Annunziata’, 87100 Cosenza, Italy; giuliana.papalia@studio.unibo.it (G.P.); gianluigi.zaza@unical.it (G.Z.); 5 Department of Nephrology and Dialysis, ASP Cosenza, 87100 Cosenza, Italy; gemmapatella@hotmail.it

**Keywords:** chronic kidney disease, kidney transplant, mediterranean diet, alkaline diet, low-protein diet

## Abstract

Chronic kidney disease (CKD) is a widespread condition with significant cardiovascular risks and a progression to end-stage kidney failure. In recent years, increasing attention has been paid to the role of dietary interventions as a factor capable of influencing disease trajectory. This review summarizes the current observational and interventional evidence on various dietary approaches in patients with CKD and kidney transplants (KTs), including Mediterranean, plant-based, and low-protein diets. A balanced Mediterranean diet, rich in fruits, vegetables, whole grains, fish, and unsaturated fats, shows promises in improving the prognosis for CKD patients. Plant-based diets, which emphasize legumes, vegetables, and grains while minimizing animal protein, improve blood pressure and the glycemic and lipid control. Low-protein diets (LPDs), typically providing less than 0.6 g/kg/day of protein, may reduce the CKD progression and nitrogen burden, further delaying the initiation of dialysis. In conclusion, diets represent a valuable and underutilized therapeutic strategy in the management of CKD and KTs, influencing disease progression and patient outcomes.

## 1. Introduction

Chronic kidney disease (CKD) is a well demonstrated pandemic syndrome affecting about 10% of the population worldwide [1]. Its onset, at the individual level, forecasts a striking risk for future cardiovascular (CV) events, kidney failure ((KF), the most advanced and dangerous phase of CKD), and overall mortality. These concepts have been well demonstrated over the past decades so that the following step was an attempt to limit the dimension of the problem. To date, significant progress has been made. At least three novel drug classes (MRA, SGLT2i, and HIF inhibitors but also GLP1-RA and ERA) have been implemented, through the conduction of phase III clinical trials, and have shown to confer protection against the aforementioned events when added to the standard of care [2,3,4]. The number of randomized studies also grew in the past few years and is going to grow in the future as well. In keeping with this, the risk stratification of patients with CKD has also been faced with an evaluation of the different prognoses by age categories, as well as by sex categories [5,6]. So, this generates the question of whether we have reached the sufficient protection of our CKD patients managed by nephrologists. In this case, in our opinion the answer is definitely no! Many problems and poor data still remain. It is emblematic that about 70% of CKD patients followed by nephrologists for at least one year still have proteinuria above its normal range and, thus, a high cardiorenal residual risk [7]. Similarly, even though the past clinical trials showed a nephroprotection provided by the novel drug classes, the absolute risk of cardiorenal events in the treatment groups is too far from being abolished. Hence, a continuous effort is needed. One old, often forgotten, therapeutic tool is represented by diet. It is demonstrated that diet is included in the lifestyle characteristics that mostly influence future prognoses, including in CKD patients [8]. In fact, a balanced diet composed of vegetables, meat, fish, and antioxidants (i.e., the Mediterranean diet) may provide a better prognosis also in patients with CKD [9]. Very few studies are available on dietary habits in kidney transplant (KT) recipients and on the interaction between diet and other variables (i.e., age, gender, and metabolic parameters) in influencing future prognoses. Moreover, randomized clinical trials (RCTs) have failed to demonstrate the value of a low-protein diet on kidney outcomes, with several concerns raised about the appropriateness of randomized studies for investigating this area [10]. Herein, with this review, we aim to summarize the current observational and intervention evidence regarding dietary approaches in patients with CKD or KTs, both of which require optimal individual risk management. The present review offers a comprehensive overview that bridges nutritional strategies and molecular insights, with a particular focus on the modulation of gut-derived metabolites, such as short-chain fatty acids (SCFAs), which may represent a mechanistic link between diet and disease progression. The relevant literature was identified through a non-systematic search of electronic databases (PubMed, Google Scholar, and Scopus), using combinations of keywords, including “chronic kidney disease”, “kidney transplant”, “mediterranean diet”, “low-protein diet”, “alkaline diet”, and “gut microbiota”. Articles were selected based on their relevance to the topic, and findings were synthesized thematically.

## 2. Biomarkers Associated with Diet

Since ancient times, as the father of medicine Hippocrates stated “Let food be thy medicine and medicine be thy food”, diet has played an undeniable role in lifestyle quality and the general health condition. Nowadays, in the specific management of CKD, nutritional treatment is recommended by the 2020 Kidney Disease Outcome Quality Initiative (KDOQI) guidelines in addition to pharmacological therapy [8]. But how and how much does diet positively influence the progression of CKD? More than 100 years ago, Addis first described the relationship between protein intake and urea excretion rates and suggested the restriction of protein intake in patients with CKD to minimize the “work overload” (and subsequent “exhaustion”) of surviving nephrons [11], posing the bases for the study of the link between diet and kidney function. Chronic kidney disease is known to cause the severe impairment of different systems, including the acid–base equilibrium, electrolyte and water balance, calcium–phosphorus metabolism, and hormonal systems [12,13], leading to oxidative stress and chronic low-grade inflammation [14].

In particular, the occurrence of metabolic complications of CKD was studied by Moranne and colleagues over 1038 CKD patients from the NephroTest cohort not on dialysis: it has been demonstrated that the prevalence of metabolic acidosis increased from 2% to 39% as the eGFR decreased from 60 to 90 mL/min/1.73 m^2^ to a value < 20 mL/min/1.73 m^2^, requiring a regular screening for metabolic disorders as the eGFR decreases below 40 mL/min/1.73 m^2^ [15]. In fact, metabolic acidosis is a consequence of the decreased overall renal excretion of acid ions, leading to a compensatory increase in acid excretion per nephron, which in turn may promote tubulointerstitial injury [16].

Acidosis actives the renin–angiotensin–aldosterone system (RAAS) with a following increased synthesis of aldosterone and increased production of endothelin, both associated with the worsening of the prognosis in terms of the progression of CKD and mortality [17]. Moreover, long-term consequences of metabolic acidosis include bone demineralization, muscular mass loss, and insulin resistance [18,19]. Thus, as a modifiable risk factor for CKD progression [20], the treatment of acidosis is needed. In order to obtain a reduction in metabolic acidosis, the KDOQI guidelines recommend an alkali treatment consisting of the oral administration of sodium citrate or sodium bicarbonate to patients with serum bicarbonate levels below 22 mmol/L [21]. However, this pharmacological intervention presents some side effects due to the sodium overload and since sodium citrate can increase gastric aluminum absorption and sodium bicarbonate can cause bloating and flatulence. Moreover, sodium contains compounds that can enhance water retention, contributing to arterial hypertension [22].

Diet has been shown to be a valid and personalized approach for this purpose since its composition influences the net endogenous acid production (NEAP, mEq/d) and, therefore, the acid–base balance of our body. NEAP results from the differences in the amounts of fixed acid (nonvolatile acid) and alkali precursors in the diet [23], and it may be measured by urine (net acid excretion, NAE) or estimated by dietary intake equations (Table 1). The potential renal acid load (PRAL) is the measurement of the capacity of the acid or alkali production of any food, and it is calculated with an equation including the amount of protein, phosphorus, potassium, magnesium, and calcium in a food [24].

Foods rich in proteins and phosphorus (i.e., meat, fish, eggs, and cheese) have a positive PRAL > 0 and are the main sources of fixed acids (amino acids) that favor the generation of hydrogen ions that increases the NEAP, while fruits and vegetables have high contents of potassium salts (negative PRAL) with alkalizing potential, favoring the production of bases and balancing hydrogen excess. Increased NEAP (which occurs in the Western diet) determines a chronic low-grade metabolic acidosis state, together with an increased risk for obesity, metabolic disease, and kidney damage [25].

The prognostic role of NEAP and the PRAL has been tested in a survival analysis to investigate their association with kidney disease. Analyzing 632 participants with CKD from the cohort from the AASK trial (African American Study of Kidney Disease and Hypertension), Scialla and colleagues have demonstrated the association between a higher NEAP dietary pattern and a faster worsening of kidney function (slope of measured GFR), especially among patients without proteinuria; while the association with renal events (doubling of creatinine or kidney failure) was not statistically significant [16]. On the other hand, a study including 642 kidney transplant recipients has demonstrated the association of a high dietary acid load with the risk of the doubling of serum creatinine or graft failure [26].

Among the variables needed to plan individualized nutrition plans for patients with CKD, there are biomarkers that directly indicate the dietary intake [27]. Among these, serum C-reactive protein and serum albumin are primarily indicators of the overall nutritional status or inflammation rather than direct indicators of a specific dietary intake. The former is considered as a good marker of cardiovascular disease; the latter has been used for decades as an indicator of malnutrition in stable conditions. Serum pre-albumin has been proposed as a more favorable nutritional marker due to its shorter half-life compared to albumin, potentially reflecting more acute changes in the nutritional state, but its levels are also significantly influenced by inflammation [28]. These laboratory measurements are part of the nutritional assessment process aimed at identifying nutritional abnormalities most frequently observed in patients with CKD: protein energy wasting syndrome, sarcopenia [29], and obesity [30].

Several laboratory measurements indicate metabolic derangements that are significantly influenced by the dietary composition, particularly in the context of CKD: hyperkaliemia and hyperphosphatasemia are both associated with a high morbidity and mortality in CKD [31] and blood bicarbonate and pH are key biomarkers for metabolic acidosis. Screening for other micronutrient deficiencies (trace elements and vitamins) may be performed based on clinical suspicion. For instance, as suggested in KDIGO guidelines [32], in patients with anemia and CKD, nephrologists should perform a basic set of measurements: complete blood count, ferritin, transferrin saturation, folate, and vitamin B12. In fact, a deficiency of these micronutrients can arise from multiple causes, including an inadequate dietary intake [33].

## 3. Evidence of the Benefits of Diet on the Progression of Chronic Kidney Disease

The Western diet is typically characterized by a high intake of saturated fats and sodium, which constitutes a high dietary acid load (50–100 mEq/daily) [34]. When kidneys cannot eliminate all of the dietary acid load due to reductions in kidney function, a state of metabolic acidosis may develop. The main dietetic schemes used to reduce the dietary acid load and to treat metabolic acidosis in CKD include the Mediterranean diet, “hypoproteic diet” and plant-based diet.

### 3.1. The Mediterranean Diet, the Possible Proper Dietary Style for Mild to Moderate Severity CKD”

The term Mediterranean diet commonly refers to the dietary pattern of populations living on the Mediterranean Sea coast (southern Italy, Greece, and southern Europe), which is characterized by a regular consumption of high alkaline-forming foods (i.e., fresh fruits, vegetables, legumes, cereals, and nuts), a moderate intake of fish and poultry, and a low intake of eggs, red meat, sweets, and dairy products [35]. The primary source of fat in the Mediterranean diet (>35% fat) is represented by olive oil, especially extra virgin olive oil (EVOO), which contains a high amount of mono- and poly-unsaturated fatty acids [36], while the moderate amount of saturated fats derives from meat and eggs. The Mediterranean diet is particularly suitable for patients with early to moderate stages of CKD, especially with cardiovascular risk factors such as diabetes, hypertension, or dyslipidemia.

The adherence to the Mediterranean diet can be assessed using specific scoring tools, such as the Mediterranean Diet Score (MDS) and the Alternate Mediterranean Diet Score (aMed), which assign scores based on the frequency of the consumption of specific food groups [37,38].

Several cohort studies demonstrated the beneficial and protective effect of the Mediterranean diet related to mortality [38], cardiovascular complications [39], and chronic kidney disease [9]. In fact, fruits and vegetables not only are important sources of nutrients, such as potassium, vitamins, and dietary fibers, but also contain phenolic compounds (especially flavonoids) which show significant bioactive antioxidant and anti-inflammatory properties [40]. Fruits are generally good sources of potassium and phosphorus, though the levels vary among different types. Bananas are particularly rich in potassium, providing about 358 mg per 100 g, while avocados offer even more at around 485 mg. Dried fruits, such as dates and apricots, have very high potassium contents—656 mg and 1162 mg per 100 g, respectively—due to their low water content. Common fruits like oranges, apples, and grapes contain moderate potassium levels, ranging from 107 to 191 mg. Phosphorus levels in fruits tend to be lower than potassium. For example, bananas contain about 22 mg of phosphorus, while apples have around 11 mg. Avocados and dried fruits again stand out, with avocados containing approximately 52 mg of phosphorus and dried apricots up to 71 mg. In general, fresh fruits provide essential nutrients and can contribute significantly to the daily potassium intake, though they are less significant sources of phosphorus compared to other food groups like dairy, meat, and legumes [41]. Also wine (in particular red wine), which is regularly consumed during meals in the Mediterranean Area and has an abundant content of resveratrol polyphenol, has been reported to have nephroprotective effects with increased kidney filtration rates [42].

Interestingly, an analysis of aMed scores calculated from the dietary data of 2403 participants with CKD from the Chronic Renal Insufficiency Cohort (CRIC) demonstrated that a higher adherence to the Mediterranean diet (higher aMed score) was associated with a lower risk of CKD progression (HR 0.75, 95% CI 0.62–0.90) compared to lower aMed scores [43].

A similar protective impact on kidney function was demonstrated by an RCT confronting Mediterranean dietary patterns versus the low-fat diet (<30% fat) in 1002 patients with type 2 diabetes mellitus and coronary heart disease from the CORDIOPREV cohort: the eGFR decline was significantly slower in the group treated with the Mediterranean diet (*p* = 0.04), especially in patients with a mildly impaired eGFR (*p* = 0.002) [44]. Moreover, a longitudinal study using three big cohorts including 3.316.633 person-years of follow-up revealed the association between the aMed and a reduced risk of developing kidney stones (HR 0.72, *p*-value < 0.001): in fact the adherence to the diet contributes to a lower urinary excretion of sodium and a higher excretion of water, citrate, acids, and magnesium, with protective effects against kidney stones [45]. Likewise, the adherence to the Mediterranean diet has been demonstrated to be associated with a better kidney graft outcome: in a study including 632 kidney transplant recipients, the aMed was inversely associated with graft failure (HR 0.68), kidney function decline (HR 0.68), and graft loss (HR 0.74), and this protective effect was more pronounced in patients with more proteinuria and with a more recent transplantation [46]. More specifically, typical Mediterranean protein sources, such as legumes and nuts, have demonstrated to be protective against CKD development, while red meat consumption is associated with CKD risk [47]. Additionally, an increased intake of fruits and vegetables has shown significant improvements in metabolic acidosis among CKD patients, although not to the same extent as oral sodium bicarbonate [48].

Clinical studies have also been performed on olive oil, the keystone of the Mediterranean diet, which has shown to possess healthy properties because of the content of polyphenols (tyrosol, hydroxytyrosol, oleuropein, secoirodoids, and lignans) [49] and mono-unsaturated fatty acids [36]. A meta-analysis of 32 cohort studies conducted by Schwingshackl and colleagues demonstrated that mono-unsaturated fatty acids from olive oil are associated with a reduced risk of stroke, CV events, and mortality [36]. A small pilot study conducted in Italy on a group of 27 patients with stage I-IV CKD revealed that the daily consumption of 40 mL of EVOO for 9 weeks was significant for the improvement of the eGFR (*p*-value 0.04) and serum concentration of albumin (*p*-value 0.021) and the reduction in the serum concentration of triglycerides (0.016) and uric acid (*p*-value 0.049) [50].

Moreover, the high intake of fibers derived from the diet improves the integrity of tight junctions in the colonic epithelium through short-chain fatty acid production and provides the modulation of the intestinal microbiota composition toward eubiosis, leading to protective effects against obesity, diabetes, cardiovascular disease, inflammation, and cancer, along with immune regulation [51]. This is of particular importance in KT recipients who are chronically exposed to immunosuppressive therapy, which is associated with an increased risk of developing hyperlipidemia, hypertension, hyperglycemia, and weight gain [52].

Despite the demonstrated advantages of a Mediterranean diet, it shows significant limitations: a diet full of fruits and vegetables is not recommended for patients with advanced stages of CKD, due to the higher risk of hyperkalemia and hyperphosphatemia; therefore, serum potassium and phosphorus levels need to be checked frequently. For a safe intake, nutritional requirements of potassium and phosphorus may be individualized according to serum levels of electrolytes, and practical counseling should be provided [53].

### 3.2. The Low-Protein Diet: An Improved CKD Progression with a Concern of Malnutrition

The low-protein diet or a very-low-protein diet have been associated with a slower progression to KF without considerable negative effects of the patient’s nutritional status in different controlled trials [54]. KDOQI guidelines suggest a low-protein diet (0.55–0.60 g/kg/day) or a very-low-protein diet (0.28–0.43 g/kg/day) with keto-analogs to reduce eGFR declines in patients with stage 3–5 CKD without diabetes and to help reduce the accumulation of nitrogenous products [55]. The rationale of a low-protein diet (LPD) is that while CKD progresses, patients accumulate nitrogen-containing products derived from protein catabolism, which progressively cause uremia and its related complications [55]. Acids derived from protein metabolism and acid retention due to kidney disease result in chronic metabolic acidosis. Moreover, a higher protein intake increases the glomerular filtration rate (GFR) through the elevation of intraglomerular pressure; overtime, this mechanism generates glomerular hyperfiltration leading to increased urinary albumin excretion [56]. Proteinuria contributes to glomerulosclerosis progression through the inhibition of podocyte regeneration [57] and can induce tubular cell apoptosis resulting in tubular atrophy [58]. Hence, it is postulated that an LPD can slow CKD progression and symptoms by reducing nitrogen waste products and lowering glomerular hyperfiltration. CKD-mineral bone disorder can also benefit from an LPD because animal proteins are a major source of phosphorous [59], thus decreasing the long-term vascular calcification.

A cost-benefit analysis of an Italian group demonstrated that a very-low-protein diet, VLPD, can delay the dialysis initiation in patients from the age of 70 years without negative effects, also providing economic advantages [60]. The LPD can also be supplemented with keto-analogs of essential amino acids, which are mandatory when a VLPD is followed to prevent malnutrition [61]. If there is an adherence to a restricted low-protein diet, keto-analogs of essential amino acids can transfer themselves into essential amino acids through the “transamination effect”, using circulating amino groups. Through this path, they can reduce the risk of malnutrition without implementing a nitrogen burden [62]. In a retrospective study published in 2023 including more than 1000 patients [63], results showed that, as compared to an LPD only, an LPD with a keto-analogs supplementation was significantly associated with a slower decline of kidney function and a lower rate of dialysis initiation. In another recent study based on the dataset of the Chang Gung Medical System of Taiwan [62], the purpose was to assess whether a low-protein diet supplemented with a keto-analog (sLPD) at a dosage of 0.6 g/kg body weight per day could decrease the risk of dialysis among patients with stage 4 CKD. Patients who subsequently underwent a ketosteril treatment (the most used keto-analog of essential amino acids) were categorized into two groups based on whether they continued the ketosteril treatment for more than three months or not. Over a one-year follow-up period, the group that continued the treatment demonstrated a significantly lower incidence of new-onset KF requiring maintenance dialysis compared to the discontinuation group. These results are encouraging; however, more research is needed to draw definitive conclusions on whether to choose a VLPD supplemented with keto-analogs (sVLPD) or an LPD only. In this regard, in another randomized controlled study [64], Bellizzi et al. compared a standard LPD to an sVLPD in patients affected by stage 4–5 CKD. Results showed patients’ poor adherence to a protein restriction diet; furthermore, there was no additional advantage of an sVLPD when compared to an LPD. In any case, in 2020 the KDOQI Guideline for Nutrition [8], a probable overall benefit of a protein restriction diet plus the supplementation of keto-analogs on RRT/renal survival in patients with stage 3–5 CKD is presumed. If we focus on patients who received a kidney transplant, an appropriate management of post-transplant diet is of major importance to improve allograft survival and outcomes [65]. A high-protein diet can induce glomerular hyperfiltration and increase the allograft’s workload, thus leading to a low nephron mass and glomerulomegaly. Overtime, this condition may result in focal segmental glomerulosclerosis [66]. Even though the literature based on a low-protein diet in KT patients is still poor. Bernardi et al. [67] demonstrated that KT recipients who were compliant with a moderate intake of protein (0.8 g/kg), sodium, and lipids maintained a stabilized kidney function during a 12 years observation when compared to non-compliant patients. The Plant-Dominant Low-Protein Diet (PLADO), which consists of an LPD with at least 50% of plant-dominant sources, has been proposed to ameliorate CKD progression both in KT and non-KT patients, and among its benefits, preventions of harmful effects of meats, a favorable effect on the microbiome, and oxidative stress reductions have been underlined [65,68]. Surely, patients and clinicians must be aware of the increased potassium load, and this diet should be thoughtfully applied to patients with advanced CKD [68]. In a recent review of dietary approaches to KT recipients [69], the potential positive impact of a vegetarian diet with a smaller animal protein intake is outlined: phosphorous from plants is less absorbed, and lower phosphoremia can be an advantage in the context of graft disfunction because of a lesser impact on vascular calcification. In the first period after the transplant though, patients often tend to exhibit hypophosphatemia [70]. Another aspect that needs to be emphasized is that KT recipients’ nutritional assessments must be carefully carried out, especially in the first year after transplantation when they tend to gain weight. For this reason, diets with a higher protein intake and low glycemic index to induce satiety sensations and weight loss are being studied [71].

What we can assume from the literature is that among patients in their early time after transplantation and patients with chronic allograft disfunction, diet management can substantially differ. Still, the overall evidence of the advantage of an LPD in slowing CKD progression and lowering the nitrogen burden is now established, and the importance of the adherence to an LPD must be stressed during clinical evaluations. Patients affected by stage 3–5 CKD should be informed by clinicians of the overall benefits of a low-protein diet, and their nutritional state should be assessed to avoid sarcopenia and malnutrition. Further studies and new criteria are needed to define whether to prescribe an LPD or an sVLPD in stage 3–5 CKD patients. Regarding kidney transplant recipients, further trials are necessary to define the appropriate protein intake, depending on graft function; the risk of diabetes; obesity; and side effects of immunosuppressive therapies, and the preservation of graft function should be considered.

### 3.3. The Plant-Based Diet: Slowing CKD Progression with the Benefits of Plants

Recently, a great interest on plant-based diets has developed worldwide. A rising body of evidence suggests that the increased consumption of plant-based foods is associated with several health benefits [72]. In fact, plant-based diets have been shown to be effective in improving blood pressure (BP), dyslipidemia, glycemic control, the body mass index (BMI), and the acid–base balance, thus lowering the risk of complications such as diabetes [73], cardiovascular disease [74], and mortality [75]. The term “plant-based” refers to a dietary pattern that focuses on foods derived primarily from plants, while limiting—but not necessarily eliminating—animal-based products. This more flexible dietary pattern differs from the traditional vegetarian diet, which is defined by the exclusion of meat and fish [76]. The plant-based diet is particularly indicated for patients in the early to moderato stages of CKD without a high risk of hyperkalemia and who prefer a vegetarian lifestyle.

Several studies documented favorable associations of plant-based diets with CKD outcomes, including the incidence (i.e., the development of albuminuria and/or eGFR declines) and progression of CKD [47,77,78]. Existing clinical trials’ data have demonstrated that the partial replacement of animal proteins with plant proteins reduces albuminuria [79,80,81]. Moreover, a recent systematic review suggested that a vegetarian diet improves renal functions in patients with CKD [82].

Three examples of plant-based dietary patterns that have been specifically designed for CKD patients not on dialysis are the alkaline diet [83] (an LPD with only 30% of proteins from animal origins), the Plant-Dominant Low-Protein Diet (PLADO) [68], and the Plant-Focused Nutrition in CKD and Diabetes Diet (PLAFOND) [84], all consisting of a protein intake of 0.6–0.8 g/kg/day from at least 50% plant-based sources.

The first condition on which the plant-based diets can demonstrate their effectiveness is the control of acidosis, one of the principal concerns in the late stages of CKD. Since the consumption of proteins leads to a higher production of acid and the consumption of fruit and vegetables leads to a higher production of alkali species, the so-called “alkaline diet” (AD) represents a nutritional approach that could be helpful for CKD metabolic acidosis management [85], as demonstrated by a series of trials. In an RCT involving 71 patients with stage 4 CKD, patients assigned to a diet including a higher fruits and vegetables intake over the course of one year had higher levels of plasma CO_2_ and lower urinary markers of kidney injury [48]. Another RCT involving 108 patients with stage 3 CKD confirmed similar effects of the fruit and vegetable intake on metabolic acidosis: the daily administration of 2 to 4 cups of fruits and vegetables over a 3-year period resulted in higher CO_2_ levels, a lower excretion of net acid, lower urinary albumin–creatinine ratios, and preserved kidney function [86]. Plant-based diets have demonstrated their efficacy in reducing inflammation and oxidative stress. Their consumption lead to a reduced generation of uremic toxins (i.e., trimethylamine n-oxide, p-cresyl sulfate, and indoxyl sulfate) [87,88] as demonstrated in two trials, respectively, on hemodialysis and non-dialysis-dependent CKD patients [89,90]. It is worth considering how, on the contrary, animal protein can promote proteolytic fermentation and subsequently results in increased indoles and phenols in the intestinal tract, decreased insulin sensitivity, and increased oxidative stress [91,92,93,94]. In addition, in the NHANES III cohort including 14,543 participants, it was observed that the dietary fiber intake was negatively associated with serum C-reactive protein (CRP) levels, such that each 10 g/day increase in the total fiber intake was associated with an 11% and 38% decline in the odds of elevated serum CRP levels in the CKD and non-CKD groups, respectively [51]

Several RCTs have shown the benefits of plant-based diets in lowering systolic BP [95,96]. The Dietary Approach to Stop Hypertension (DASH) trial showed that the DASH diet (a largely plant-based diet) reduced BP by 5.5 mmHg compared to the control diet [97]. A meta-analysis performed using data from seven RCTs involving 313 participants confirmed similar benefits: a mean reduction in systolic BP by 4.8 mmHg was observed in patients adhering to vegetarian diets compared to omnivorous diets [98]. Through various pathways, different components of vegetarian diets contribute to a lower BP in CKD, directly or indirectly. First, unprocessed plant-based foods contain less sodium than processed or animal-based foods. A lower sodium intake can prevent and control hypertension and lower albuminuria levels, as demonstrated by two meta-analyses including, respectively, dialysis patients and kidney transplant recipients [99] and patients with stage 1–4 CKD [100]. Second, the dietary fiber intake improves hypertension by its effect on arterial contraction, influencing the angiotensin-converting enzyme (ACE) activity and retaining electrolytes (such as potassium and magnesium) in its matrix [8,101]. In addition, plant-based diets may be effective for the prevention and treatment of diabetes mellitus. During digestion, soluble dietary fibers increase viscosity, resulting in the trapping of carbohydrates, slowing the absorption of glucose, and lowering the postprandial glycemia. A dietary fiber intake, in addition to clinical benefits already reported in Section 3.1, is also able to delay gastric emptying, improving insulin sensitivity and producing greater satiety [84,102].

It is useful to underline that a common concern with plant-based diets is the over consumption or over-accumulation of minerals, such as potassium, phosphorus, and sodium, that are contraindicated in CKD patients. Despite the fact that these minerals tend to be in higher abundances in vegetables, they tend to be less bioavailable compared to in animal-based foods due to a reduced absorption of naturally found minerals from plant sources. In fact, potassium contained in fruits and vegetables is typically ingested with non-chloride anions and bicarbonate that promote an increased urinary potassium excretion [103]. However, when hyperkalemia occurs, possible treatments include fighting constipation and binding potassium in the colon with the use of oral binders or increasing the potassium excretion in the urine with the prescription of drugs, such as diuretics or SGLT2i. [104]. Both of the newer potassium binders (patiromer and sodium zirconium cyclosilicate SZC) have shown to lower potassium levels in hyperkalemic CKD patients, even in those receiving RAAS inhibitors [105,106]. These newer agents have shown improved safety profiles: sodium and calcium polystyrene sulfonate (SPS) binders were effective but associated with potentially serious adverse events for the digestive system, such as intestinal necrosis. The most common gastrointestinal events reported with patiromer were mild to moderate constipation, diarrhea, and nausea, each occurring in approximately 4% of patients [106]. Studies have shown that SZC can rapidly reduce serum potassium to normal levels within 48 h, so physicians can prescribe it in cases of acute hyperkalemia [107]. Moreover, as reported in the APPETIZE study, both patiromer and SZC outperformed the sodium and calcium polystyrene sulfonate binder on overall palatability, an essential feature of a drug for optimal compliance [108].

Phosphorus from plant-based foods has also proven to be less bioavailable (20–40% bioavailability) compared to animal foods and processed foods, which mostly have phosphorus in the form of caseins (40–60% bioavailability) and food additives (~100% bioavailability), respectively [95,96]. The reduced phosphorus bioavailability from plant-based foods is due to the phosphorus primarily bound to phytate, which is poorly absorbed by the gastrointestinal tract given humans’ lack of phytase enzymes in the gut [95,97,98,99,100].

Potential drawbacks and limitations to a plant-based diet must be considered. Generally, plant-based diets are considered to be healthier; however, this dietary pattern is typically deficient in vitamin B12, of which animal-based diets are instead rich in. In the setting of a plant-based diet, taking supplements of vitamin B12 is essential for people at risk of deficiency, including those following vegan diets [109] and patients with CKD who follow low-protein diets, have a compromised absorption of nutrients, and take medications (e.g., metformin and proton-pump inhibitors) that can reduce the assimilation of vitamin B12 [110]. Another limitation is the potential nutritional inadequacy of plant-based diets in people with CKD, who are more predisposed to malnutrition–wasting conditions (protein-energy wasting), that have adverse impacts on health and survival [111]. Protein sources considered as high-quality are so defined if they contain easily digestible and absorbable essential amino acids and in quantities adequate to support human growth [112].

The currently recommended method for evaluating dietary protein quality is the amino acid scoring system, which considers most animal proteins and soy proteins to be complete protein sources [112]. Although individual plant-based proteins (with the exception of soy protein) have insufficient levels of one or more indispensable amino acid, the consumption of different sources of plant-based proteins can help to reach the adequate intake of indispensable amino acids, providing health benefits [113]. Several studies in experimental animal models [114,115,116] and in humans [117] have shown that plant-based diets are indeed nutritionally adequate in CKD. For example, it was reported that vegetarian diets with very low protein contents (0.3 g/kg/day) supplemented with keto-analogs provided an adequate nutritional status (i.e., serum albumin levels and the BMI remained stable over 29.6 months) [118]. Additionally, a systematic review including 141 observational and interventional studies from Europe, South-East Asia, and North America reported that the average protein intake was lower in patients consuming plant-based diets compared to those consuming animal-based diets, but the overall dietary protein intake was well within the recommended intake levels for both groups, and the dietary energy intake was comparable among those receiving plant-based vs. animal-based diets [119]. Limited studies among non-CKD [120] and CKD populations [121] have reported that a higher consumption of fruit and vegetables was correlated with a reduced risk of sarcopenia. Future studies evaluating the impact of animal-based diets vs. plant-based diets on muscle heath are needed, with a consideration of the energy intake and overall diet quality.

In order to optimize a plant-based diet intake, it is appropriate for nephrologists to provide CKD patients with some advice: the consumption of vegetables with a negative PRAL (kale, broccoli, Brussels sprouts, cabbage, onions, garlic, celery, zucchini, lettuce, cucumber, radish, bell pepper, rocket, and sprouted seeds); the inclusion of two portions (about 250 g per day) of those vegetables in two meals per day; and two portions of fruit (about 300 g per day) according to their serum potassium content. Some vegetables are naturally rich in potassium, and spinach is among the top sources. It provides about 558 mg of potassium and 49 mg of phosphorus per 100 g. Potatoes—especially when boiled—offer approximately 379 mg of potassium and 44 mg of phosphorus per 100 g. Cooked beet greens are exceptionally high in potassium, delivering around 909 mg, along with 73 mg of phosphorus. Vegetables that are moderate in their potassium content (between 150 and 200 mg per 100 g) include cauliflower, carrots, cabbage, broccoli, and zucchini. Low-potassium vegetables (less than 150 mg per 100 g) include the following: cucumbers, with about 147 mg of potassium and 24 mg of phosphorus; iceberg lettuce, with around 141 mg of potassium and 20 mg of phosphorus; and onions, with about 146 mg of potassium and 29 mg of phosphorus. For patients with hyperkalemia and hyperphosphatemia, soaking and boiling can help lower the potassium and phosphorus in foods [122]. The consumption of legumes, such as lentils, beans, and chickpeas, as an alternative source of protein instead of meat is recommended because of their low PRAL, as well as the consumption of meal/grain foods such as bread, breakfast cereals, rice, and pasta. However, the consumption of these foods should be adjusted to avoid exceeding the daily caloric intake (30–35 kcal/kg/day in CKD patients) [123,124] and, as a consequence, weight gain [18].

## 4. Shared Aspects of Diet Between CKD and Kidney Transplants

The nutritional status represents an important factor in a patient’s prognosis after a kidney transplantation, and nutritional behaviors can have an important influence on patient morbidity and graft survival [125]. The assessment of the nutritional status is an important component of the transplant candidate work-up [126]. An unbalanced nutritional status predisposes some patients to develop post-transplant complications, such as increased blood pressure, elevated lipid levels, disturbances in glucose metabolism, and weight gain [127]. These complications represent risk factors for cardiovascular disease, which constitutes one of the main causes of death in patients after kidney transplantations [128].

Two distinct phases of nutritional changes are in the early and late post-transplant period [129]. Most transplant candidates are dialysis patients or some pre-dialytic patients with KF. For this, the major nutritional and metabolic problems of the early post-transplant phase are malnutrition, protein energy wasting, low albumin, and a low body mass index (associated or not with systemic inflammation) [130,131,132]. Obesity is far more prevalent than undernutrition in most transplant programs and is also well known to adversely impact post-transplant outcomes [132]. In addition, high doses of corticosteroids, prescribed in the early post-transplant phase, may rapidly produce a moon face, truncal obesity, lipid abnormalities, glucose intolerance, hypertension, as well as calcium, phosphorus, and vitamin D imbalances [133]. Limiting the carbohydrate intake by up to 50% of the total calories, fractionated in several meals, seems appropriate to avoid hyperglycemia and also possibly to avoid cushingoid effects of corticosteroids [133].

In general, the recovery of renal function observed immediately after a successful renal transplantation is followed by an overall improvement in the nutritional status [134]. In fact, changes in the body composition after a KT are due to the increase in appetite and the reduction in uremic toxins and substances derived from the chronic inflammation in KF, as well as to the immunosuppressive treatment [134,135,136,137,138]. After a KT it has been estimated that patients gain around 10% to 35% of their weight, and most of the weight gain takes place in the first 3 years after transplantation when new dietary habits are included [71,128,139,140]. The most important factors implied in the weight gain in the KT population are the immunosuppressive regimen, the cessation of dietary restrictions associated with dialysis, appetite restoration, and improvements in quality of life [141,142]. The resultant overweight or obesity could lead to the development of insulin resistance and, as a consequence, may lead to diabetes mellitus in the long term after a KT [137,143,144]. Many authors emphasize the significance of persistent weight gain and obesity in the long-term post-transplant period and difficulties in weight reduction [132,145,146]. As a result, dietary restrictions should be applied to avoid a rapid weight gain and the resulting complications, as reported in various studies [138,147]. For this reason, international clinical practice guidelines on the evaluation and care of kidney recipients recommend screening for weight gain or obesity by means of anthropometric and biochemical parameter tests [126,148]. A clinical study by Kluch M et al. [149] has evaluated 154 patients’ nutritional intake and described the results of nutritional trends in patients over the long term after KTs. As resulted, the amount of the protein, cholesterol, sugar, phosphorus, and sodium intake in patients after KTs exceeds recommended norms for the daily intake in the general population. Studies exploring interventions to promote weight loss or maintenance in kidney transplant recipients are still scarce [136,150,151]. Due to the lack of high-quality evidence on this issue, there are no guidelines or recommendations for a specific nutritional intervention to manage weight gain and obesity after a kidney transplantation [8,152,153].

New-onset diabetes after transplant (NODAT) develops in 10–45% of kidney transplant recipients [154,155]. Dysglycemia is almost universal in the first weeks after transplantation: insulin resistance is promoted by postsurgical inflammation, and high-dose immunosuppressive medications promote insulin resistance; in addition, patients receiving tacrolimus present an impaired insulin production [154,156]. Furthermore, the restoration of the kidney function enhances insulin clearance [154,155].

The use of cyclosporin rather than tacrolimus [156] and a reduction in total steroid exposure [157] have been shown to reduce the incidence of NODAT and the requirement for insulin post-transplant, and a rapid steroid withdrawal was found to reduce insulin requirements though not the diabetes prevalence [158].

Post-transplant diabetes and an impaired glucose tolerance after transplantation have been associated with significantly increased risks of major adverse cardiovascular events and cardiovascular mortality [159,160]. Here, the use of hypoglycemic medication coupled with lifestyle and dietary advice represent the most effective strategy [161]. Evidence exists to support the adoption of a Mediterranean-style diet to reduce the incidence of both metabolic syndrome [162] and NODAT [163].

A further finding is that after KTs patients have an elevated sodium intake [164,165] compared to the recommended daily sodium intake of 1500 mg per day [166]. Furthermore, a correlation between exceeding the daily recommended sodium intake and the occurrence of hypertension in transplant recipients treated with cyclosporine has been reported [167]. In addition, a sodium-rich diet could have a negative effect on the kidney graft function and an indirect influence on the carbohydrate and lipid metabolism, as well as the development of obesity [125,127,139,168].

With regard to protein intake, during the post-transplant period, the amount of protein consumption should not exceed the recommended daily dose of 0.73 ± 0.11 g per kilogram (g/kg) of body weight as in the general population [169]. This may safely stabilize the GFR and slow the progression to renal failure by reducing the proteinuria [149]. A moderate protein intake (0.7 g/kg) in the early period post-transplant is recommended to match protein catabolism, increase muscle mass, and improve the course of chronic rejection and reduce mortality risk [170]. However, data on the impact of protein intake on long-term outcomes in kidney transplant recipients are scarce [67,171,172]. Bernardi et al. evaluated a low-protein, low-lipid, and low-sodium diet in a 12-year follow-up study and showed a protective effect for the kidney with this dietary regimen [67], but the interpretation of the results is limited and controversial. Van Den Berg et al. [171] studied the association between protein intake and blood pressure, proteinuria, and creatinine clearance in a cross-sectional study with 625 renal transplant recipients, and no deleterious effects of the diet were identified. For all intents and purposes, it would be appropriate to consider that, unlike the initial post-KT phases, a restriction in protein intake may be a useful strategy in slowing the progression of renal disease in chronic rejection [173].

The calcium, phosphorus, and vitamin D metabolism are influenced by several factors, including necrosis, fractures, and bone mass loss due to prolonged therapy with steroids leading to osteopenia and osteonecrosis, as well as an incomplete renal functional restoration after transplantation [174]. The daily recommendation for the dietary calcium intake is about 800 to 1500 mg. Calcium supplements may be indicated when dietary calcium does not compensate for hypocalcemia [129]. On the other hand, cyclosporine use is associated with an incidence of hyperkalemia, especially during the early post-transplant phase when the dosage is higher [129]. Additionally, antihypertensive agents, such as beta-blockers or conversion enzyme inhibitors, may exacerbate the hyperkalemia. In the case of hyperkalemia or oliguria, the dietary restriction of potassium to 1 to 3 g per day (g/d) may be indicated [129].

An increased phosphorus concentration in a patient’s blood after a KT can promote graft failure [175]. On the other hand, hypophosphatemia is very common during the early weeks post-transplant as a result of phosphaturia; in this case, supplementation may be needed [176]. The daily recommended phosphorus intake should be from 1200 to 1500 mg per day (mg/d), as hypophosphatemia may persist indefinitely for most renal recipients. Some patients may also require phosphorus supplementation, and some may require a supplementation of active vitamin D in doses of 1 to 2 g/d [129].

Several studies showed that early dietary guidance and appropriate nutritional management have a protective effect regarding metabolic complications related to dietary regimens in KT patients [136,177].

It is important to consider that the gut microbiota exerts a significant influence on the modulation of immunological, metabolic, renal, and cardiovascular health [178]. Kidney transplantation is a condition that has been shown to disrupt the gut microbiota [179]. Dysbiosis in transplant recipients is manifested with the reduction in microbial diversity with the increase in potentially pathogenic gut bacteria, including the colonization with multidrug resistant bacteria [179]. The proposed mechanisms to explain the dysbiosis of the gut microbial community include polypharmacy, medical comorbidities, the transplant surgery itself, as well as the induced immune suppressive state [180].

Short-chain fatty acids (SCFAs) are produced by the bacterial digestion of dietary complex carbohydrates that cannot be digested by humans [179]. SCFAs may act locally as a food substrate for “good” bacteria [179]. The consumption of a Mediterranean diet, typically high in fruit, vegetables, whole grains, dietary fiber, and fat-reduced dairy products and low in processed foods and animal meat, was shown to be beneficial in the long-term function of kidney allografts [181]. Such benefits were thought to be mediated by anti-inflammatory effects of N-3 Poly-Unsaturated Fatty Acids; however, the restoration of the gut microbiota and the enhancement of SCFA production may be an additional mechanism of benefit [179].

A healthy, balanced diet should be diverse and provide essential nutritional and energetic ingredients [135,182,183,184].

Non-adherence to nutrition therapies is common among renal transplant recipients [164,185] comparable to patients with CKD [186]. The implementation of strategies aimed at improving patients’ compliance with dietary prescriptions after transplantation could enhance their quality of life and long-term prognosis.

## 5. Molecular Aspects of Research Around Diet and Kidney Transplants

Despite improvements in the care of KT recipients, outcomes remain suboptimal, especially for long-term graft survival. The KT outcome is affected by numerous immunological and donor-related factors. However, the lifestyle and dietary regimen are strong drivers of comorbidities, including cardiovascular disease, obesity, and diabetes [69]. Even though the Mediterranean and Dietary Adherence to Stop Hypertension (DASH) dietary patterns have been demonstrated to be the most advantageous dietary patterns for KT recipients [69], very little molecular evidence has been described.

In a recent study, Heldal et al. reported a statistically significant association between death-censored long-term kidney graft loss and patterns of systemic inflammation in the early period after kidney transplantation, including fibrogenesis activity and metabolic and general/vascular inflammation [187].

Interestingly, fibrogenesis activity markers, like growth differentiation factor 15 (GDF-15) and Cathepsin S, have been described as reduced in subjects who followed the Mediterranean or Healthy Nordic Diet [188].

Several metabolic pathways can be impaired after a KT, leading to pathological states like obesity, hyperglycemia, dyslipidemia, and hypertension [189]; nevertheless, still very little has been described about dietary regimen effects. Among possible biomarkers, resistin, a hormone secreted from adipose tissue, has been correlated with metabolic disorders, atherosclerosis, and inflammation [190]. Data reported by Nagy and colleagues have shown associations between serum resistin levels and clinical outcomes, such as mortality and graft loss, in a large KT recipient cohort [191]. Serum resistin levels have also been positively correlated with saturated fat intake and inversely associated with an adherence to the Mediterranean diet [192].

The immune response towards the allogeneic organ contributes to low-grade persistent inflammation in KT recipients, which may be mirrored at the systemic level and increase mortality risks [193]. C-reactive protein (CRP) is a molecule largely associated with inflammation and cardiovascular disease [194], and over the past few decades its consistently high concentration in plasma and sera in KT recipients has been described as a biomarker for renal rejection [195]. In a systematic review and meta-analysis of randomized controlled trials published by Koleman et al., they recapitulate the recent evidence on the effects of different dietary patterns on inflammatory and immune-related biomarkers in humans, and CRP has been shown to be significantly diminished in subjects who followed the Mediterranean, DASH, or vegetarian/vegan diet [196].

Cytokines such as Interleukin-8 (IL-8), Interleukin-6 (IL-6), and tumor necrosis factor-α (TNF-α) are markers of inflammation, and increased levels of these cytokines in urine and serum have been significantly associated with allograft rejections in KT patients [197]. Furthermore, Interferon-gamma inducible protein-10 (IP-10/CXCL10) and Monocyte Chemoattractant Protein-1 (MCP-1/CCL2) are chemokines that play a role in the alloimmune response against kidney allografts, and high urinary levels have been associated with rejection [198]. Intriguingly, several studies have shown that the Mediterranean regimen appeared to be the most prominent dietary pattern associated with reductions in inflammatory biomarkers, such as IL-8, IL-6, TNF-α, IFN-ɣ, and MCP-1 [196]. IL-18, a pro-inflammatory cytokine associated with a greater risk of cardiovascular events, graft failure and mortality, has also been described to be significantly reduced after a long-term adherence to the Mediterranean diet + EVOO [199].

Additionally, while transplantation leads to metabolic alterations in the immune system, especially on T cells [200], the nutritional approach, like caloric restriction or intermittent fasting, can deeply remodel immune cell niches [201]. Nevertheless, if and how specific/personalized dietary patterns can be effective in improving graft tolerance and all the comorbidities needs to be further investigated on a molecular level.

Recently, more and more studies are also reporting a correlation between KT outcomes and the gut microbiota, since the latter has been shown to play a role in alloimmunity, infections, and the immunosuppressant and antibiotics metabolism [202]; beneficial effects seem to come from a dietary fiber intake [203].

In KT recipients, dysbiosis—affected by immunosuppressive therapies, antibiotics, lifestyle, and diet—alters the gut microbiota composition, typically increasing *Firmicutes*, Proteobacteria, and Verrucomicrobia while reducing Bacteroidetes and Actinobacteria [204,205,206,207]. The microbial shift impairs the intestinal barrier, increasing permeability and bacterial translocation [208,209]. This activates pro-inflammatory pathways, including the cytokine release (IL-1, IL-6, IL-18) and Th1/Th17 cell differentiation, while reducing regulatory T cells and promoting graft rejection via immune cross-reactivity [210]. Dysbiosis is associated with acute rejections, infections, fibrosis, diarrhea, altered drug levels, and a reduced SCFA production [211]. Gut-derived uremic toxins (e.g., TMAO, p-cresyl sulfate) further exacerbate renal inflammation and damage [212].

SCFAs exert anti-inflammatory and metabolic effects via G-protein-coupled receptors (GPR41, GPR43, GPR109A), supporting glucose regulation, atherosclerosis prevention, and immune tolerance [211,213,214]. Conversely, TMAO promotes atherogenesis, monocyte activation, inflammasome signaling, an impaired cholesterol metabolism, and an increased thrombosis risk [215].

Diet strongly influences microbial health: Western and high-protein diets promote dysbiosis and inflammation by reducing SCFA-producing bacteria (e.g., *Roseburia*), lowering fecal butyrate levels, and increasing TMAO [216,217,218]. Conversely, plant-based, DASH, and Mediterranean diets lower the Firmicutes/Bacteroidetes ratio; increase SCFAs; reduce TMAO; and improve blood pressure, lipid profiles, and the immune balance [219,220,221,222,223].

The gut microbiota should be considered a new frontier to modulate and personalize therapies and nutritional approaches to improve graft survival and patients’ quality of life.

All aspects considered need further studies to clarify the molecular aspects and define biomarkers linking dietary regimens and outcomes in the transplant patient. In addition, an appropriate stratification based on immunosuppressive therapies is required.

## 6. Lifestyle Differences Between Men and Women with Chronic Kidney Disease: The Role of Diet

Sex-specific differences in genetics, physiology, and immunology as well as gender factors (i.e., how the individual self-identifies and behaves) influence the kidney pathophysiology and disease course. Globally, the prevalence of CKD-ND according to the eGFR is greater in women than men, while there are conflicting data about the impact of sex-based factors on the progression of CKD. The variability of these data could reflect differences in the kidney disease etiology and outcomes [224].

A meta-analysis from J. Neugarten et al. based on 68 studies and a total of 11,345 patients with CKD from different causes observed a more rapid decline in renal function with time in men than women. However, this analysis cannot assess whether this result is independent from other covariates, such as diet, blood pressure, or serum lipid levels [225].

In the univariate analysis of The Modification of Diet in Renal Disease Study, a prospective multicenter study, male gender was identified as a risk factor for a more rapid progression of the eGFR decline in 840 primarily nondiabetic subjects with CKD. However, the multivariate analysis showed that only proteinuria, high-density lipoprotein (HDL) levels, and BP are independent risk factors for a worse renal outcome [226].

More recently, Minutolo et al., with their multicohort study considering 2335 patients affected by moderate to advanced CKD, confirmed the impact of sex on the progression of kidney disease, reporting a 50% higher risk of progression in men than in women [5].

Even data from the initial 10 years of the Swedish Renal Registry–Chronic Kidney Disease, a nationwide, population-based inception registry of CKD G3b-G5 patients, reported an overall rate of CKD progression of 19.6% per year, with men showing a higher risk of progression and of death, especially from cardiovascular disease [227].

The biological mechanisms at the basis of men’s faster decline of the eGFR is not known. Among the more reliable theories there is the possible impact of sex-based differences in oxidative stress, nitrogen oxide metabolism, and sex steroids [228].

Animal studies, for example, have shown a protective and anti-inflammatory activity of estrogens on podocytes. However, Swartling O. et al. found no differences between the decline of the eGFR in women than in men before and after the mean menopausal age in Sweden [229].

All these data could be affected by an epidemiological bias as confirmed by data from population-based studies that have shown that women are more represented than men in earlier stages of CKD [230].

Minutolo et al. suggested that the fastest renal progression in men could, in part, be related to higher levels of proteinuria in men compared with women [5].

What we would speculate is that even diet could have affected the rate of the progression of the kidney damage. Gender differences on dietary choices impact the protein burden and the creation of a pro-inflammatory environment, with men preferring a high-protein diet and women preferring an antioxidant-rich diet. However, more studies need to be performed.

## 7. Social, Cultural, and Economic Factors Influencing Dietary Adherence

Dietary adherence is fundamental for slowing the progression of CKD, managing uremic symptoms, and preventing complications such as electrolyte imbalances and cardiovascular disease [8]. However, it is strongly influenced by social, cultural, and economic factors. Family and social support can facilitate adherence, particularly in patients who struggle with motivation or meal preparation. When family eating habits align with CKD dietary restrictions, adherence becomes less challenging. Changes in lifestyle may be required to stick to regular meal schedules and avoid fasting routines. Also, the patient’s cultural background must be taken into consideration for appropriate communication to improve understanding and compliance: traditional foods may provide high contents of sodium, potassium, and phosphorus and may conflict with dietary restrictions; religious practices or dietary laws (e.g., halal and vegetarianism) may be adjusted to prioritize nutrition advice; and if a language barrier is present, it must be addressed to avoid misinterpretation and confusion. In low-resource settings, access to fresh and affordable foods should not be taken for granted: renal-friendly foods are often more expensive than processed alternatives and many patients may find it difficult to afford recommended foods, such as fresh fruits and vegetables. Food supply constraints, such as living in food deserts with limited transportation possibilities, further restrict access to suitable dietary choices.

Social determinants of health play a crucial role in dietary adherence, and addressing these determinants is essential for improving patient outcomes.

In our digital era, technology can be a useful tool in dietary management: there are mobile applications that can remind users of eating schedules or that reinforce positive behaviors towards food by texting messages to patients. Online groups with other patients can be an opportunity to exchange recipes or to find solutions for overcoming barriers to adhering to the diet or to agree on group workouts. Telemedicine could increase the patient’s adherence to the diet by online follow-up appointments or telephone calls. Technology can also assist nephrologists in detecting and monitoring sarcopenia in CKD patients: there are imaging techniques (such as ultrasound or computed tomography) that allow for a non-invasive and thorough evaluation of muscle quality, including the assessment of ectopic fat infiltration [231].

## 8. Conclusions

**Figure 1:**
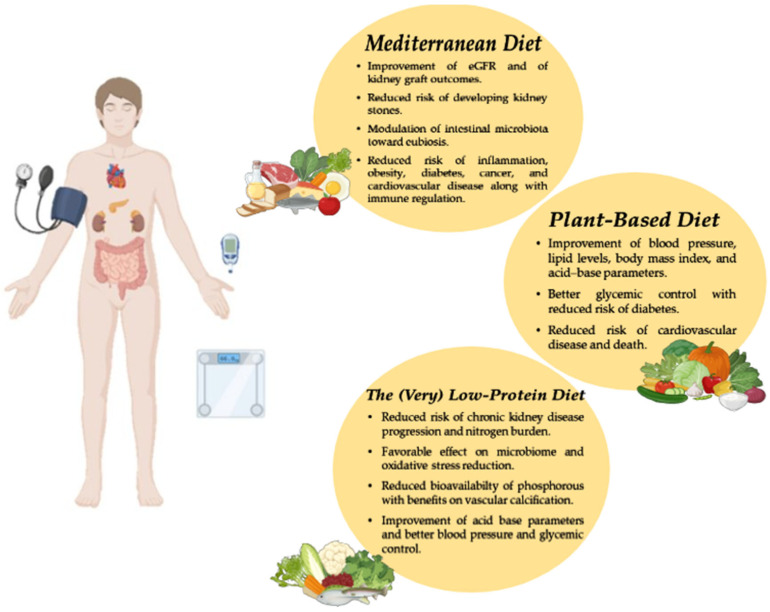
Impact of Mediterranean, plant-based, and low-protein diets in improving kidney disease outcomes.

Managing diets is a therapeutic tool that can influence the prognosis of CKD patients (Figure 1 and Table 2). Several dietary patterns have been shown to offer health benefits to CKD patients; therefore, an individualized dietary plan should be developed based on each patient’s nutritional needs and food preferences. Given the progressive and chronic nature of CKD, a periodic reassessment is essential to further personalize the dietary plan to the patient’s evolving clinical needs and to ensure nutritional adequacy. Moreover, nephrologists should educate patients on lifestyle modifications as a strategy to delay CKD progression and improve cardiovascular outcomes, alongside blood pressure control and the use of new drug classes. By integrating clinical and molecular perspectives, this review may serve as a foundation for future research and dietary interventions in nephrology.

## Figures and Tables

**Table 1:** NEAP and PRAL equations. NAE, net acid excretion. NEAP, net endogenous acid production. OAes, organic acid excretions. PRAL, potential renal acid load.

NEAP (mEq/d) = 54.5 × (protein intake [g/d] ÷ potassium intake [mEq/d]) − 10.2
OAes (mEq/d) = body surface area × 41/1.73
PRAL = 0.49 × protein (g/d) + 0.037 × phosphorus (mg/d) − 0.021 × potassium (mg/d) − 0.026 × magnesium (mg/d) − 0.013 × calcium (mg/d)
NAE = urinary titratable acid + urinary ammonia nitrogen − excretion of filtered HCO^3−^
NAE = PRAL + OAes

**Table 2:** The main diets for CKD patients.

	Key Messages
Mediterranean Diet	- A balanced Mediterranean diet may improve the prognosis for individuals with CKD [29,31,39]; - It is characterized by high consumption of alkaline-forming foods such as fresh fruits, vegetables, legumes, cereals, and nuts [26]; - It includes moderate intake of fish and poultry and low intake of eggs, red meat, sweets, and dairy products [27]; - The primary source of fat is olive oil, especially EVOO which is high in mono- and poly-unsaturated fatty acids [27]; - Limitations include the risk of hyperkalemia and hyperphosphatemia in advanced stages of CKD due to high fruit and vegetable intake [43].
The Low-Protein Diet	- LPD reduces CKD progression and nitrogen burden, further delaying dialysis initiation [50]; - KDOQI guidelines suggest a low-protein diet (0.55–0.60 g/kg/day) or a very-low-protein diet (0.28–0.43 g/kg/day) with keto-analogs to reduce eGFR decline in patients with stage 3–5 CKD without diabetes [45]; - LPD can be supplemented with keto-analogs of essential amino acids, which can reduce the risk of malnutrition without increasing nitrogen burden; - Limitations include malnutrition, the difficulty of maintaining low-protein diet, and the increased potassium load, requiring careful application in patients with advanced CKD [55].
The Plant-Based Diet	- It has been shown to be effective in improving blood pressure (BP), glycemic control, lipid levels, and body mass index (BMI), thus lowering the risk of complications such as diabetes, cardiovascular disease, and death [82,89,90]; - It is associated with favorable CKD outcomes, including incident CKD and CKD progression [66,67]; - It can control acidosis by promoting higher production of alkali species [72]; - It may have potential nutritional inadequacy of vitamin B12 and protein contents [98,99]; - The “plant-based” adjective can be applied to some diets such as the Plant-Dominant Low-Protein Diet (PLADO) and the Plant-Focused Nutrition in CKD and Diabetes Diet (PLAFOND) and alkaline diet (only 30% proteins found in animal products) [58]; - Limitations include deficiency in vitamin B12 and potential nutritional inadequacy.

## Data Availability

No new data were created or analyzed in this study. Data sharing is not applicable to this article.

## Abbreviations

The following abbreviations are used in this manuscript:

BP	blood pressure
CKD	chronic kidney disease
CRP	C-reactive protein
CV	cardiovascular
DASH	Dietary Adherence to Stop Hypertension
EVOO	extra virgin olive oil
GFR	glomerular filtration rate
IL-8, IL-6	Interleukin-8, Interleukin-6
IP-10	Interferon-gamma inducible protein-10
KT	kidney transplant
LPD	low-protein diet
MCP-1	Monocyte Chemoattractant Protein-1
NEAP	net endogenous acid production
NODAT	new-onset diabetes after transplant
PLADO	Plant-Dominant Low-Protein Diet
PLAFOND	Plant-Focused Nutrition in CKD and Diabetes Diet
PRAL	potential renal acid load
SCFAs	short-chain fatty acids
sVLPD	VLPD supplemented with keto-analogs
TNF-α	tumor necrosis factor-α
VLPD	very-low-protein diet
